# Selanylimidazopyridine Prevents Lipopolysaccharide-Induced Depressive-Like Behavior in Mice by Targeting Neurotrophins and Inflammatory/Oxidative Mediators

**DOI:** 10.3389/fnins.2018.00486

**Published:** 2018-07-19

**Authors:** Micaela Domingues, Angela M. Casaril, Paloma T. Birmann, Darling de A. Lourenço, Beatriz Vieira, Karine Begnini, Eder J. Lenardão, Tiago Collares, Fabiana K. Seixas, Lucielli Savegnago

**Affiliations:** ^1^Programa de Pós-Graduação em Biotecnologia, Grupo de Pesquisa em Neurobiotecnologia, Biotecnologia/Centro de Desenvolvimento Tecnológico, Universidade Federal de Pelotas, Pelotas, Brazil; ^2^Programa de Pós-Graduação em Química, Laboratório de Síntese Orgânica Limpa, Química/Centro de Ciências Químicas, Farmacêuticas e de Alimentos, Universidade Federal de Pelotas, Pelotas, Brazil; ^3^Programa de Pós-Graduação em Biotecnologia, Grupo de Pesquisa em Oncologia Celular e Molecular, Laboratório de Biotecnologia do Cancer, Biotecnologia/Centro de Desenvolvimento Tecnológico, Universidade Federal de Pelotas, Pelotas, Brazil

**Keywords:** major depressive disorder, lipopolysaccharide, imidazopyridines, selenium, depression, NFkB, mouse models of depressive disorder, neuroinflammation

## Abstract

Inasmuch, as the major depressive disorder (MDD) has been characterized as a heterogeneous disease as the inflammatory processes, neurotrophic factors’ dysfunction and oxidative/nitrosative stress are believed to play a vital role in its establishment. Organoselenium compounds stand out due to their antioxidant, anti-inflammatory, neuroprotective, and antidepressant effects. In this sense, the present study investigated the effect of 3-((4-methoxyphenyl)selanyl)-2-phenylimidazo[1,2-a]pyridine (MPI; 20 and 50 mg/kg, intragastrically) pretreatment [30 min prior lipopolysaccharide (LPS) challenge (0.83 mg/kg)] on acute LPS induced depressive-like behavior, neuroinflammation, and oxidative stress. MPI was able to prevent the increased immobility time induced by LPS on the forced swimming test (FST), the increase in pro-inflammatory cytokines’ expression in the hippocampus (HC) of mice after LPS challenge via NFkB downregulation, and the increase of the reactive oxygen species generation and lipid peroxidation in the prefrontal cortex and HC of mice. It was observed that at the doses tested, MPI protected against reducing levels of BDNF in the cortex and HC of mice challenged with LPS. These observations suggest that the antidepressant-like effect of MPI depends on its capacity to modulate the inflammatory, antioxidant, and neurotrophic systems.

## Introduction

Major depressive disorder (MDD) is a serious illness with great loss in quality of life, increased morbidity and mortality, and a high rate of recurrence and chronicity (Réus et al., 2016). For many years, the pathophysiology of MDD has been focused on the monoaminergic theory, proposing that depression is caused by a decreased monoaminergic function in the brain (Maes et al., 2011). Although the monoaminergic systems are clearly involved in the etiology of depression, it is now accepted that inflammatory processes, neurotrophic factors’ dysfunction, and oxidative/nitrosative stress might also be involved in the establishment of this disorder (Maes et al., 2013). Based on these observations, rodent models of inflammation-associated depression have been developed, such as administration of bacterial lipopolysaccharide (LPS), which is a potent activator of the immune system (Dantzer et al., 2008).

Classic antidepressants, which are effective in around 30–40% of the patients, (Moreno et al., 1999), are often associated with numerous side effects, which compromise their acceptance and use by patients (Wang et al., 2016). Hence, in recent years, there has been a growing search for new molecules which display antidepressant activity with multitarget characteristics. In this context, the selenium-containing compounds have received attention, since these molecules demonstrate an amplitude of biological properties (da Rocha et al., 2012). Among the biological functions, they have been widely explored in the literature as anti-inflammatory, antioxidant, and antidepressant (Tiegs et al., 1998; Bortolatto et al., 2013; Pinto Brod et al., 2016). Donato et al. (2015) demonstrated the antidepressant-like effect of an organoselenium molecule in the tail suspension test (TST) through modulation of the noradrenergic and dopaminergic systems. Besides, Casaril et al. (2017) showed the anti-neuroinflammatory effect of a selenium-containing indolyl compound. In this sense, normalization of activated inflammation pathways may be a novel target for the discovery of new antidepressant molecules (Stefânia et al., 2013). In addition to the organoselenium compounds, the imidazopyridines have been found to possess interesting biological properties (Casaril et al., 2017). These molecules have been used to treat insomnia, and have shown promising antioxidant, anti-inflammatory, and neurotrophic effects (Parekh et al., 2013; Dymińska, 2015; Qian et al., 2016). Considering the pharmacological proprieties of organoselenium compounds and imidazopyridines, the combination of both molecules may be a relevant strategy for the development of more efficient drugs with multitarget profile for the treatment of depression.

We reported in this study that 3-((4-methoxyphenyl)selenyl)-2-phenylimidazo[1,2-a]pyridine (MPI) mitigates the LPS-induced depressive-like behavior and investigated the mechanisms of this molecule in the biomarkers of oxidative stress, neuroinflammation, and neurotrophic factor.

## Materials and Methods

### Animals

The experiments were conducted in male Swiss mice (25–30 g). Six animals per box were kept under standard environmental conditions (24 ± 1°C and light/dark cycles of 12 h) with free access to water and food. All procedures were performed according to the guidelines of the Ethics Committee on Animal Experimentation of the Federal University of Pelotas (CEEA/UFPel-1870-2016). All efforts were made to minimize animals suffering and to reduce the number of animals used in the experiments.

### Drugs

**FIGURE 1:**
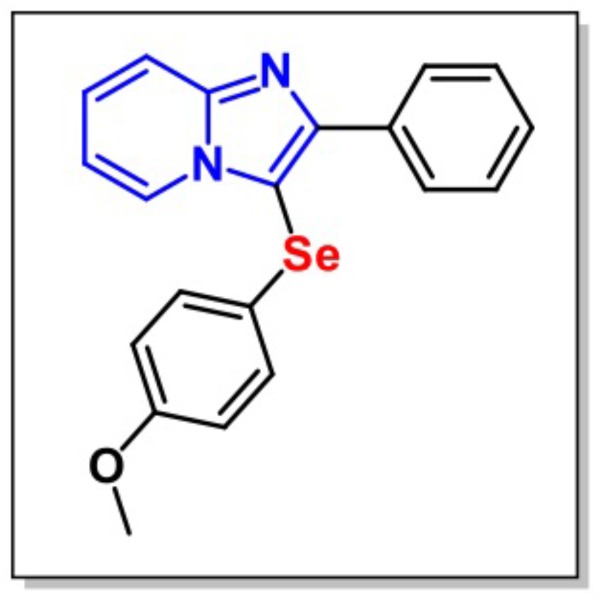
Chemical structure of 3-((4-methoxyphenyl)selanyl)-2-phenylimidazo[1,2-a]pyridine (MPI).

3-((4-Methoxyphenyl)selenyl)-2-phenylimidazo[1,2-*a*]pyridine (**Figure 1**) was synthesized by the Laboratory of Clean Organic Synthesis (LASOL-UFPel). *Escherichia coli* LPS (L-3129, serotype 0127:B8) was purchased from Sigma–Aldrich Co. (St. Louis, MO, United States). MPI was diluted in canola oil (a non-polar and inert substance) and administered intragastrically (i.g.) at a constant volume of 10 ml/kg body weight. LPS was diluted in saline at a constant dose of 0.83 mg/kg and administered intraperitoneally (i.p.). Drug solutions were prepared freshly in the morning. All other chemicals used in the present study were of analytical grade. Appropriate vehicle-treated groups were simultaneously assessed.

### Experimental Procedure

Animals were randomly divided into six experimental groups, each group consisting of six mice (*n* = 6). The groups were used as follows: Group I: canola oil was administered 30 min prior to saline injection. This group served as a normal control group; Group II: Canola oil was administered 30 min prior to LPS injection. This group served as a disease control group; Group III: MPI (20 mg/kg, i.g.) was administered 30 min prior to saline injection; Group IV: MPI (50 mg/kg, i.g.) was administered 30 min prior to saline injection; Group V: MPI (20 mg/kg, i.g.) was administered 30 min prior to LPS injection; and Group VI: MPI (50 mg/kg, i.g.) was administered 30 min prior to LPS injection. The different doses of MPI (20 and 50 mg/kg) were selected from previous literature evaluating organoselenium compounds’ antidepressant-like effects in mice (Gerzson et al., 2012; Casaril et al., 2015; Donato et al., 2015).

The LPS dose was chosen based on previous studies (O’Connor et al., 2009). After 24 h of LPS or saline administration, depressive-like behavior was evaluated. Behavior tests were carried out by a trained observer blind to the treatments. The behavior analyses, open field test (OFT), and forced swimming test (FST) were assessed after 24 h of the LPS or saline injection (Zhang et al., 2016). All the behavioral tests were made in all the animals. The sequence of the protocol was OFT–FST (Martinez et al., 2014).

The cytokines and neurochemical determinations were evaluated by taking different animals (following the same experimental design) in order to avoid interferences from behavioral assessment on neurochemical parameters (Casaril et al., 2017). It is important to notice that the mice were anesthetized by isoflurane before the sacrifice. They were then killed by cervical dislocation, followed by brain removal and isolation of prefrontal cortex (PFC) and hippocampus (HC) for analysis. The brain tissues were removed and the right hemisphere was used to measure cytokines (TNF-α, IL-1β), NF-κB (using a primer sequence corresponding to subunit p65), and BDNF expression, while the left hemisphere was used to determinate reactive oxygen species (ROS) formation and lipid peroxidation.

### Open Field Test (OFT)

Open field test was the first behavioral test performed. Before all the treatments, we determined the baseline between all the groups. After this first phase, we submitted the animals to the respectives treatments and the OFT was performed 24 h after LPS or saline administration. This procedure evaluates the possible effects of the compound, whether it interferes in the locomotor and exploratory activity of the animals. Briefly, the mice were placed in the center of a wooden box (30 cm × 30 cm× 15 cm) divided into nine squares of equal areas, and during 5 min, the locomotor (through the number of crossed squares) and exploratory (number of elevations) activities were evaluated.

### Forced Swimming Test (FST)

Forced swimming test is used to evaluate the immobility time as the absence of escape-oriented behavior, which is an important symptom of depression (Porsolt et al., 1977). In this test, mice were individually placed into a cylinder (25 cm height, 10 cm diameter) containing 8 cm of water maintained at 25°C. After a habituation period (2 min), the immobility time (s) of the animals was rated for a further 4 min time. This test was performed by two experienced raters, who were blind to the treatment group.

### Neurochemical Determinations

The animals were euthanized 24 h after administration of saline and LPS. The brains were dissected for the removal of HC and PFC. The left hemispheres were homogenized in Tris-HCl (50 mM, pH 7.4; 1:10, w/v). The homogenate was centrifuged at 2500 × *g* for 10 min at 4°C, and a low-speed supernatant fraction (S_1_) was used for the measuring of the thiobarbituric acid reactive species (TBARS) levels and determination of ROS. The right hemispheres were immersed in Trizol, stored at -80°C, and used for the qRT-PCR assay.

### Evaluation of Lipid Peroxidation

The lipid peroxidation in the cerebral structures (PFC and HC) was evaluated by measuring the thiobarbituric acid (TBA) reacting substances as an index of RS (Ohkawa et al., 1979). Briefly, S_1_ was incubated with 8.1% sodium dodecyl sulfate (SDS), 0.8% TBA and acetic acid/HCl (pH 3.4) at 95°C during a period of 2 h. Lipid peroxidation was detected by the absorbance at 532 nm in a spectrophotometer. The results were expressed as nmol MDA/g tissue.

### Reactive Oxygen Species (ROS) Quantification

The ROS levels formed in PFC and HC of the left hemisphere were determined by conventional spectrofluorimetry using the dichloro-dihydro-fluorescein diacetate (DCHF-DA) reagent (Loetchutinat et al., 2005). Briefly, DA-DCHF (1 mM) was incubated together with S_1_ and Tris-HCl buffer (10 mM, pH 7.4). The oxidation of DCHF to fluorescent dichlorofluorescein (DCF) was measured for intracellular RS detection. The fluorescence intensity is measured with emission at 520 nm and excitation at 488 nm in spectrofluorimeter and the results are expressed in units of fluorescence.

### RNA Extraction and Gene Expression Evaluation by qRT-PCR

Total RNA was purified from right hemispheres PFC and HC using Trizol reagent (Invitrogen^TM^, Carlsbad, CA, United States). cDNA synthesis was accomplished with 1 μg RNA using the High Capacity cDNA Reverse Transcription kit (Applied Biosystems^TM^, United Kingdom) according to the manufacturer’s protocol. SYBR Green PCR Master Mix (Applied Biosystems^TM^, United Kingdom) was used for performing Real-time qRT-PCR, according to the manufacturer’s protocol. Relative values of gene expression were normalized using GAPDH and the conditions for the reaction included 95°C for 15 s, 60°C for 60 s, and 72°C for 30 s. The 2^−ΔΔCT^ (Delta–Delta Comparative Threshold) method was used to normalize the fold change in gene expressions. Primer sequences and full name of the genes are provided in **Table 1**. It is importante to notice that p65 NFκB analyzed was obtained in the whole tissue, which is a limitation in this study.

**Table 1:** Primers’ sequences.

Gene	Sequência 5′–3′
GAPDH	S-AGGTCGGTGTGAACGGATTTG
	A-TGTAGACCATGTAGTTGAGGTCA
BDNF	S-CCATAAGGACGCGGACTTGTAC
	A-AGACATGTTTGCGGCATCCAGG
TNF-α	S-CATCTTCTCAAAATTCGAGTGACAA
	A-TGGGAGTAGACAAGGTACAACCC
IL-1β	S-CTGTGTCTTTCCCGTGGACC
	A-CAGCTCATATGGGTCCGACA
NFκB p65	S-GCT TTC GCA GGA GCA TTA AC
	A-CCT TTC GCA GGA GCA TCA AC

### Statistical Analysis

Data from behavior and neurochemical analysis were expressed as mean ± SEM (standard errors of the mean). The results were analyzed by one-way analysis of variance (ANOVA) followed by the Student–Newman–Keuls test for *post hoc* comparison. Results were considered significant (*P* < 0.05). The statistical analysis was accomplished by using Graph Pad Prism version 7.0 for mac, Graph pad Software (San Diego, CA, United States).

## Results

### Effects of Pretreatment With MPI on the Psycholocomotor Activity

**FIGURE 2:**
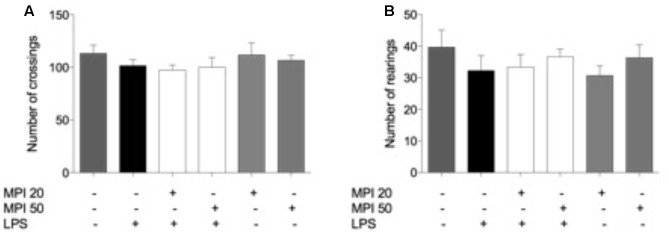
Effects of MPI on the OFT induced by LPS. **(A)** Number of crossings and **(B)** rearings in OFT. Data are shown as mean ± SEM. ^###^ *p* < 0.001 when compared to the control group. ^∗∗∗^*p* < 0.001 when compared to LPS-induced group (*n* = 6 for each group).

In order to exclude possible inhibitory or excitatory effects of LPS and MPI, the number of crossings and rearings was measured in the OFT before FST. It is importante to notice that the baseline was analyzed before the treatments and no significant diferences were observed in the groups analyzed in the number of crossing [*F*(5,30) = 0.9765, *p* = 0.4480] and number of rearing [*F*(5,30) = 1.492, *p* = 0.222] (data not shown). As shown in **Figures 2A,B**, the treatments did not produce significant differences in the number of rearing [*F*(5,30) = 0.7789, *p* = 0.57] and crossing [*F*(5,30) = 0.5698, *p* = 0.72]. These results indicate that LPS and MPI did not cause alterations in the psychomotor and exploratory activity of the animals.

### Pretreatment With MPI Prevented the Increase in the Immobility Time Induced by LPS

**FIGURE 3:**
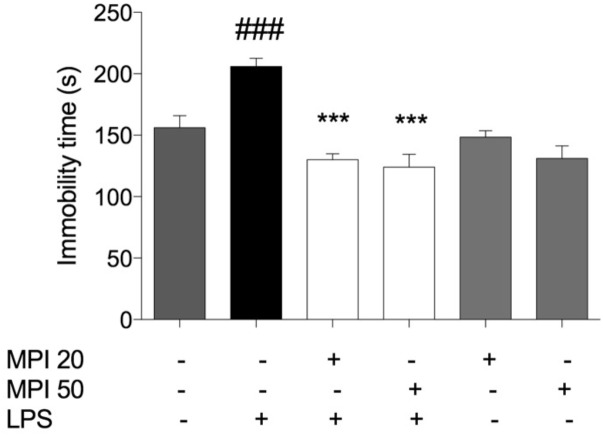
Effects of MPI on the depressive-like behaviors induced by LPS. After 24 h of LPS administration, the LPS-induced group had significantly increased the immobility time measured in the FST compared to the control group (*F*_5,30_ = 12.22, *P* < 0.001, *post hoc* ^###^*P* < 0.001, *n* = 6) (###). MPI followed by LPS administration prevented the increase in the immobility time when compared to the LPS-induced group (*F*_5,30_ = 12.22, *P* < 0.001, *post hoc* ^∗∗∗^*P* < 0.001, *n* = 6) (^∗∗∗^). No significant differences observed among the control and MPI followed by saline admnistration. Data were analyzed using a one-way ANOVA followed by the Student–Newman–Keuls multiple comparisons *post hoc* test. Error bars represent SEM.

Effect of MPI on the immobility time determined in FST is depicted in **Figure 3**. The systemic administration of LPS caused a significant increase in the duration of the immobility time when compared to saline-treated mice (control group; *P* < 0.001). However, pretreatment with MPI (20–50 mg/kg) prevented the increase in the immobility time induced by LPS (*P* < 0.001), suggesting an antidepressant-like effect of the compound. Pretreatment with MPI (20 and 50 mg/kg) followed by administration of saline indicated no change when compared to saline-treated mice.

### Pretreatment With MPI Prevented Lipid Peroxidation Induced by LPS in PFC and HC of Mice

**FIGURE 4:**
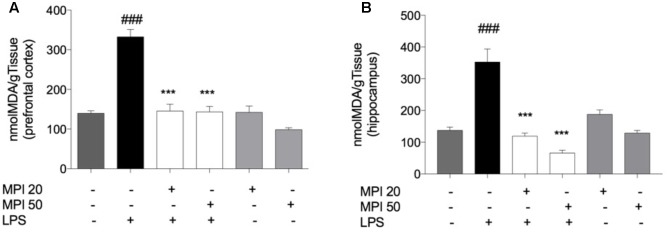
Effect of MPI on lipid peroxidation in LPS-challenged mice. After 24 h of LPS administration, the LPS-induced group had significantly increased the TBARS levels in PFC **(A)** and HC **(B)** ^###^*P* < 0.001 when compared to the control group. MPI followed by LPS administration prevented the increase of TBARS levels in PFC (*F*_5,30_ = 35.47, *P* < 0.001, *post hoc* ^∗∗∗^*P* < 0.001, *n* = 6) and HC (*F*_5,30_ = 26.18, *P* < 0.001, *post hoc* ^∗∗∗^*P* < 0.001, *n* = 6) (^∗∗∗^) when compared to the LPS-induced group. No significant differences observed among the control and MPI followed by saline admnistration. Data were analyzed using a one-way ANOVA followed by the Student–Newman–Keuls multiple comparisons *post hoc* test. Error bars represent SEM.

Lipopolysaccharide challenge induced significant increase in levels of lipid peroxidation in the PFC and HC (*P* < 0.001; **Figures 4A,B**). Pretreatment with MPI (20 and 50 mg/kg) prevented lipid peroxidation induced by LPS in the PFC (*P* < 0.0001) and HC (*P* < 0.001). However, administration of the compound alone did not influence lipid oxidation, maintaining similar values to the control group.

### Pretreatment With MPI Prevented the RS Formation Induced by LPS in PFC and HC of Mice

**FIGURE 5:**
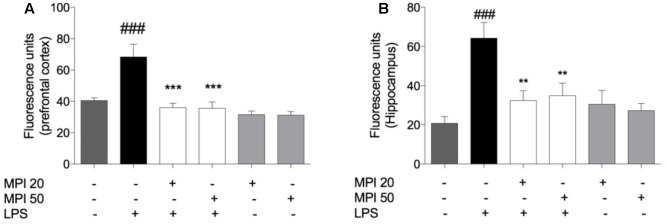
Effect of MPI on RS formation in LPS-challenged mice. After 24 h of LPS administration, the LPS-induced group had significantly increased the RS formation in PFC **(A)** and HC **(B)** ^###^*P* < 0.001 when compared to the control group. MPI followed by LPS administration prevented the increase of RS formation in PFC (*F*_5,30_ = 4.472, *P* < 0.001 *post hoc* ^∗∗∗^*P* < 0.001, *n* = 6) (^∗∗∗^) and HC (*F*_5,30_ = 6.317, *p* < 0.01, *post hoc* ^∗∗^*P* < 0.01, *n* = 6) (^∗∗^) when compared to the LPS-induced group. No significant differences observed among the control and MPI followed by saline admnistration. Data were analyzed using a one-way ANOVA followed by the Student–Newman–Keuls multiple comparisons *post hoc* test. Error bars represent SEM.

The level of RS formed in the PFC (*P* < 0.001) and HC (*P* < 0.001) of mice is expressed in **Figures 5A,B**. LPS challenged-mice showed significant increase in the RS production in PFC and HC, when compared to the control group. Noteworthy, the pretreatment with MPI (20 and 50 mg/kg) prevented the RS formation induced by endotoxin in the PFC (*P* < 0.001) and HC (*P* < 0.001). In addition, the pretreatment by MPI, followed by saline administration, maintained similar values to the control (saline).

### MPI Prevented the Pro-inflammatory Cytokine Production Induced by LPS in the PFC and HC of Mice

**FIGURE 6:**
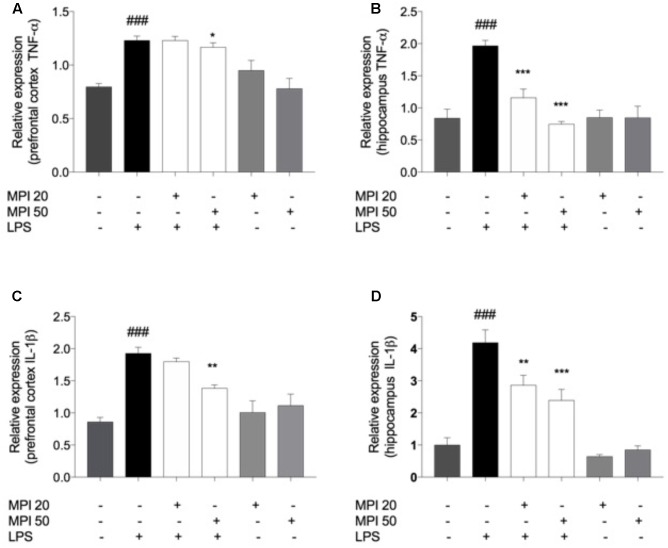
TNF-α levels in **(A)** PFC and **(B)** HC and IL-1β in PFC **(C)** and **(D)** HC of LPS-induced group mice increased significantly when compared to the control group ^###^*P* < 0.001. MPI followed by LPS administration prevented the increase of TNF-α levels in **(A)** PFC only in the 50 mg/kg dose (*F*_5,30_ = 10.06, *P* < 0.05 *post hoc* ^∗^*P* < 0.05, *n* = 6) (^∗^) and in the both doses in **(B)** HC (*F*_5,30_ = 16.69, *P* < 0.001, *post hoc* ^∗∗∗^*P* < 0.001, *n* = 6) (^∗∗∗^) when compared to the LPS-induced group. MPI followed by LPS administration prevented the increase of IL-1β expression in **(C)** PFC only in the 50 mg/kg dose (*F*_5,30_ = 13.21, *P* < 0.05 *post hoc* ^∗^*P* < 0.05, *n* = 6) (^∗^) and in the both doses in **(D)** HC (*F*_5,30_ = 23.63, *P* < 0.001, *post hoc* ^∗∗∗^*P* < 0.001,^∗∗^*P* < 0.01, *n* = 6) (^∗∗∗^)(^∗∗^) when compared to the LPS-induced group. No significant differences observed among the control and MPI followed by saline admnistration. Data were analyzed using a one-way ANOVA followed by the Student–Newman–Keuls multiple comparisons *post hoc* test. Error bars represent SEM.

In order to investigate the mechanism underlying the behavioral changes induced by LPS, the levels of pro-inflammatory cytokines IL-1β and TNF-α were evaluated (**Figure 6**). LPS challenge caused a significant increase of cytokines levels in the PFC and HC, when compared to the control (*P* < 0.001). The increase of TNF-α (**Figure 4B**) was prevented by pretreatment with MPI at the highest dose in the PFC (*P* < 0.05) and in both doses in the HC (*P* < 0.001). MPI in both doses was capable of preveting IL-1β expression in the HC (*P* < 0.001; **Figure 6D**) and in PFC (**Figure 6C**) at the highest dose (50 mg/kg; *P* < 0.05; **Figure 6C**).

### MPI Prevented the Increase of NFκB Levels in the PFC and HC of Mice

**FIGURE 7:**
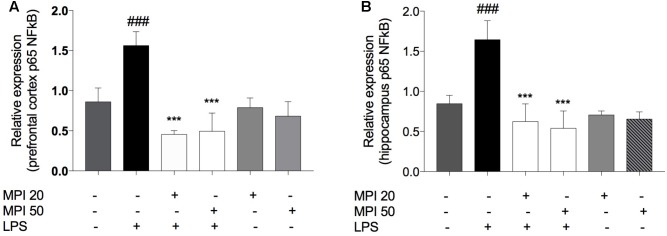
p65 NFκB expression in **(A)** PFC and **(B)** HC of LPS-induced group mice increased significantly when compared to the control group ^###^*P* < 0.001. MPI (20–50 mg/kg) followed by LPS administration prevented the increase of NFκB expression in **(A)** PFC (*F*_5,30_ = 30.45, *P* < 0.001 *post hoc* ^∗∗∗^*P* < 0.001, *n* = 6) and in **(B)** HC (*F*_5,30_ = 31.82, *P* < 0.001, *post hoc* ^∗∗∗^*P* < 0.001, *n* = 6) (^∗∗∗^) when compared to the LPS-induced group. No significant differences observed among the control and MPI followed by saline admnistration. Data were analyzed using a one-way ANOVA followed by the Student–Newman–Keuls multiple comparisons *post hoc* test. Error bars represent SEM.

Lipopolysaccharide significantly increased NFκB levels compared to control in PFC (*P* < 0.001) and HC (*P* < 0.001). MPI (20 and 50 mg/kg) significantly prevented the increase of this transcription factor, both in the PFC and in the HC (*P* < 0.001 and *P* < 0.001; **Figures 7A,B**). Administration of MPI alone did not cause any change in baseline neurochemical parameters.

### MPI Prevented the Decrease in BDNF Levels in the PFC and HC of Mice Due to LPS Administration

**FIGURE 8:**
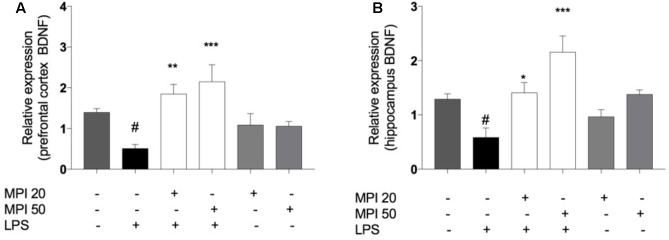
BDNF expression in **(A)** PFC and **(B)** HC of LPS-induced group mice decreased significantly when compared to the control group ^###^*P* < 0.001. MPI (20–50 mg/kg) followed by LPS administration prevented the decrease of BDNF expression in **(A)** PFC (*F*_5,30_ = 8.751, *P* < 0.001; *post hoc* ^∗∗^*P* < 0.01, ^∗∗∗^*P* < 0.001, *n* = 6) and in **(B)** HC (*F*_5,30_ = 6.775, *P* < 0.001, *post hoc* ^∗^*P* < 0.05,^∗∗∗^*P* < 0.001, *n* = 6) (^∗∗∗^)(^∗∗^) when compared to the LPS-induced group. ^#^*P* < 0.05 when compared to the control group. No significant differences observed among the control and MPI followed by saline admnistration. Data were analyzed using a one-way ANOVA followed by the Student–Newman–Keuls multiple comparisons *post hoc* test. Error bars represent SEM.

Lipopolysaccharide significantly reduced BDNF levels compared to control in PFC, (*P* < 0.001) and HC (*P* < 0.001). MPI (20 and 50 mg/kg) significantly prevented the reduction of this neurotrophic factor, in the PFC (*P* < 0.01 and *P* < 0.001; **Figure 8A**) and in the HC (*P* < 0.05 and *P* < 0.001; **Figure 8B**). Administration of MPI alone did not cause any change in baseline BDNF levels.

## Discussion

In this study, we have demonstrated the antidepressant-like effect of MPI, a novel multitarget compound, in the LPS-induced depressive-like behavior. This effect was accompanied by the prevention of LPS-induced NFκB activation and pro-inflammatory cytokines (TNF-α and IL-1β) expression in the brain. Besides, our study has also demonstrated that the antidepressant-like effect of MPI might be related with its antioxidant properties, since it prevented the LPS-induced oxidative damage by reducing the ROS formation and lipid peroxidation in PFC and HC. Another major finding was that MPI was able to prevent the LPS-induced BDNF downregulation, by increasing its expression in PFC and HC of mice.

The observed effects of MPI may be related to its chemical structure, which combines selenium and imidazopyridine. Organoselenium compounds can exert a variety of biological actions, including anti-inflammatory, neuroprotective, antioxidant, and antidepressant activities (da Rocha et al., 2012; Pinto Brod et al., 2016; Casaril et al., 2017). Similarly, imidazopyridines also present several pharmacological properties, such as anti-inflammatory (Qian et al., 2016), antiviral, antioxidant, and neuroprotective (Dymińska, 2015) activities. These multimodal pharmacological effects provided a rationale for the study of their multitarget action to treat MDD (Jiang et al., 2016). Considering that MDD is accompanied by activation of immuno-inflammatory pathways, oxidative stress, and BDNF reduction, the combination of both pharmacophore groups in MPI can be a promising alternative to treat this disorder (Maes et al., 2012; Moylan et al., 2013; Slyepchenko et al., 2016).

Lipopolysaccharide administration is a widely accepted model to investigate the link between depressive-like behavior and related neurochemical alterations (Ferreira Mello et al., 2013). This occurs because this endotoxin is recognized by the Toll-like receptor 4 (TLR-4), thereby inducing neurochemical changes in rodent brains, such as increase of oxidative stress, pro-inflammatory cytokines expression, and the decrease in BDNF levels, through the NFκB pathway activation. The LPS administration increases peripheral cytokines and leads to sickness behaviors, including a decrease in food and water intake and lack of movement. Around 24 h postinjection, mice begin eating and moving identically to control animals, as well as their sick behaviors, have resolved. Pro-inflammatory cytokines’ increased levels lead to an elevation of indoleamine 2,3 dioxygenase (IDO) 24 h after LPS injection. During the same time that IDO is elevated, animals will present depressive-like behavioral changes including anhedonia and spend more time immobile in the FST (O’Connor et al., 2009). Regarding depressive-like behavior induced by LPS 24 h after its administration, antidepressant and anti-inflammatory treatments can reverse or prevent this condition (Yirmiya, 1996; Ohgi et al., 2013).

The results of our study remained consistent with previous ones since the increase of immobility time in FST was prevented by pretreatment with MPI at doses of 20 and 50 mg/kg, indicating the antidepressant-like effect of this compound. It is worth noting that administration of MPI alone did not cause a significant change when compared to the control group, being effective only in the disease condition. In addition, MPI has not influenced the locomotor and exploratory activity in mice and this result is in accordance with previous works which have demonstrated that 24 h after injection of LPS the motor activity is back to normal when compared to the other groups. These results exclude the possibility that the FST results were affected by alterations in locomotor activity.

MPI antidepressant-like effect was accompanied by the restoration of LPS-induced alterations of inflammatory mediators (i.e., TNF-α and IL-1β), neurotrophic factors through the NFκB downregulation. Moreover, MPI decreased lipid peroxidation and ROS formation in PFC and HC. Slyepchenko et al. (2016) demonstrated that increased oxidative stress could induce neuroinflammation and contribute to the development of depression. Maes et al. (2011) also demonstrated that peripherally administered cytokines could reach the brain and lead the immune activation and ROS production via NFκB pathway. Once in the brain, these mediators can modulate the release of monoamines, by activation of the tryptophan-kynurenine pathway-cytokine induced, enhancing the re-uptake of monoamines and decreasing the concentration of the cofactor tetrahydrobiopterin (BH4), thereby reducing the synthesis of monoamines. Besides we analyzed the levels of p65 NFκB, it was obtained in the whole tissue in both structure of mice, which is a limitation in this study.

In this study, the antidepressant-like effects of MPI were accompanied by decreasing TNF-α and IL-1β levels in brain areas related to mood regulation. There is significant evidence that both cytokines play important roles in modulating mood behavior (Drevets et al., 2008). Interestingly, IL-1β plays a pivotal role of microglial activation induced by repeated stress, since the genetic deletion of IL-1β mitigates microglial activation induced by social defeat stress (Wohleb et al., 2011). Zhang et al. (2016) showed that IL-1β impairs the differentiation of hippocampal neural progenitor cells into serotonergic neurons indicating the antidepressant effect of IL-1β antagonists. The pro-inflammatory cytokine TNF-α also contributes to the pathogenesis of depression by an activation of neuronal serotonin transporters, stimulation of indoleamine 2,3-dioxygenase, and activation of hypothalamic-pituitary-adrenocortical (HPA) axis (Bossù et al., 2012; Zhang et al., 2016). Additionally, classically activated macrophages (M1) pro-inflammatory cells are activated by LPS, while alternatively activated macrophages (M2) exert an anti-inflammatory effect, and act by reducing the neuroinflammation (Slyepchenko et al., 2016). Accordingly, a recent study has found evidence that selenium supplementation increases the polarization of macrophages from M1 to M2-like phenotype, decreasing neuroinflammation (Kudva et al., 2015), suggesting that the antidepressant effect of MPI associated to its anti-inflammatory activity can be related to the presence of selenium in its structure.

Lipopolysaccharide alone decreased the levels of BDNF by increasing the levels of pro-inflammatory cytokines. Several reports demonstrated that LPS-induced cytokines expression decreased BDNF and others neurotrophins expression. Previous studies have shown that LPS has the potential to cause cognitive dysfunction by this mechanism.

Besides the above-mentioned effects of cytokines in the brain, it is known that these pro-inflammatory mediators can reduce the expression of BDNF. The signaling pathway of this neurotrophic factor plays an important role in brain neuroplasticity and depression pathogenesis (Lee and Kim, 2010). Peripheral administration of LPS reduces the amount of BDNF in the brain (Guan and Fang, 2006), which was prevented in MPI-treated mice. Accumulated evidences point to a reduction in the HC size in depressive patients (Eyre and Baune, 2012; Slyepchenko et al., 2016). It is known that BDNF levels are much higher in HC when compared to other brain structures, due to the great biological importance of the HC in memory maintenance and involvement with emotions (Eyre and Baune, 2012). Studies have shown that neuroinflammation interferes in the generation of new neurons, since the systemic administration of LPS, IL-1β, and TNF-α contributes to the reduction of BDNF expression (Kohman and Rhodes, 2014). Our results showed that MPI prevented the changes in BDNF levels induced by LPS administration, when compared to the LPS group. We assumed that MPI prevented the BDNF reduction since MPI was also able to prevent the increase of pro-inflammatory cytokines expression in the rodent brains. Of note, the expression of the pro-inflammatory cytokines may be dependent on the NFκB expression, as also demonstrated in the present study. These results show us a new perspective about the effect of MPI, since BDNF is involved with cognition control and depressive patients present potential cognitive dysfunction, due to BDNF levels decrease. Interestingly, the HC is the brain region that has the highest recruitment of cells of the immune system after some kind of stress, setting up a specific response to the stressor, reducing the levels of these pro-inflammatory cytokines (Brachman et al., 2015). However, the compound did not show effect in TNF-α levels in PFC, indicating its anti-inflammatory effect mainly in HC, main brain area associated to MDD. Thus, it may be suggested that the compound acts as an immunomodulator, increasing the recruitment of M2 cells to HC, in order to diminish the damage caused by LPS administration, by preventing the increase of IL-1β and TNF-α levels in this brain area through reduction of NFκB expression. Moreover, BDNF and its interaction with ROS may play a role in several symptoms of neuropsychiatric abnormalities. The downregulation of BDNF increases the vulnerability to oxidative damage under stressful circumstances (Hacioglu et al., 2016). The oxidative stress plays an important role in the neurobiology of MDD (Tomaz et al., 2014; Liu et al., 2015; Casaril et al., 2017). High metabolic rate and an abundance of peroxide substrates make the brain cells more vulnerable to oxidative damage. Various studies have showed that depressed patients present higher levels of oxidative stress biomarkers such as malondialdehyde (Painsipp et al., 2011; Tomaz et al., 2014; Casaril et al., 2017). It is known that high levels of this biomarker lead to mitochondrial dysfunction causing cell death, biomolecules damage, and more ROS production. Interestingly, lipid peroxidation products decrease CREB-dependent BDNF promoter activity in rat hippocampal neurons. Thus, products of lipid peroxidation are associated to BDNF and related cognitive function (Pugazhenthi et al., 2006). In addition, excess of ROS also influences the depletion of monoamines in the synaptic cleft, through the oxidation of BH4 (Miller and Raison, 2016). Peripheral immune activation by LPS causes change in the brain’s redox system, by increasing the ROS directly by activating microglia (Maes et al., 2012; Liu et al., 2015; Slyepchenko et al., 2016). Exposure to high doses of LPS triggering prolonged production of cytokines which lead to increase of free radicals production might cause deleterious condition termed oxidative stress (O’Connor et al., 2009). ROS are involved in the mechanism of LPS toxicity, in particular in NFκB activation. In general, there is a balance between ROS generation and antioxidant defenses in cells. When ROS generation overcomes the cellular antioxidants, oxidative stress is generated and its beneficial functions might harm the host leading to neuroprogression of MDD. Cytokines increased levels induced by LPS administration lead to NFκB activation which contributes to upregulation of NADPH oxidase (NOx) and inducible nitric oxide synthase (iNOS) and consequently culminate in the free radicals raise.

In the present study, MPI significantly prevented the increase of ROS production in brain areas, demonstrated by the quantification of ROS by the use of DCFH-DA (Loetchutinat et al., 2005) indicating the antioxidant profile of the compound. Considering that LPS increases lipid peroxidation as a consequence of oxidative stress, in the present study, MDA levels, measured by TBARS (Ohkawa et al., 1979), were prevented by MPI in HC and PFC. This suggests that this molecule also prevents lipid peroxidation caused by the oxidative condition. In this view, it can be stated that the compound has antioxidant activity, suggesting that the antidepressant-like effect may also be due to this property. Considering that the compound presented protective effect against NF-kB upregulation induced by LPS, the neuroprotector effect of the compound may be associated with its anti-inflammatory effect.

Current treatments are estimated to only reduce about one-third of the disease burden of depressive disorders, in this sense the preventive alternatives may be a strategy to treat this progressive disease since it causes several brain changes. Hence, pretreatment with MPI is a promising strategy to further reduce the disease burden of depression, due to its several proprieties.

## Conclusion

To sum up, the present study demonstrated that the antidepressant effect of MPI at doses of 20 and 50 mg/kg might be related, in part, to its ability to modulate the oxidative, immune-inflammatory pathways and restore BDNF levels, altered by LPS induction. These multitarget proprieties make MPI a promising treatment to MDD. However, more studies are needed to accurately understand the molecular basis of the antidepressant activity of this molecule, and then progress to clinical studies.

## Author Contributions

MD and AC designed the study and the protocol, managed literature searches and manuscript writing, and collected, assembled, analyzed, and interpreted data with LS, PB, DL, KB, FS, and TC. BV and EL synthetized and characterized the organoselenium compound. All authors revised the manuscript and approved the final manuscript.

## Conflict of Interest Statement

The authors declare that the research was conducted in the absence of any commercial or financial relationships that could be construed as a potential conflict of interest. The reviewer GA and handling Editor declared their shared affiliation.
